# Aromatase inhibitors, estrogens and musculoskeletal pain: estrogen-dependent T-cell leukemia 1A (*TCL1A*) gene-mediated regulation of cytokine expression

**DOI:** 10.1186/bcr3137

**Published:** 2012-03-09

**Authors:** Mohan Liu, Liewei Wang, Tim Bongartz, John R Hawse, Svetomir N Markovic, Daniel J Schaid, Taisei Mushiroda, Michiaki Kubo, Yusuke Nakamura, Naoyuki Kamatani, Paul E Goss, James N Ingle, Richard M Weinshilboum

**Affiliations:** 1 Division of Clinical Pharmacology, Department of Molecular Pharmacology and Experimental Therapeutics, Mayo Clinic, 200 First Street SW, Rochester, MN 55905, USA; 2 Division of Rheumatology, Department of Medicine, Mayo Clinic, 200 First Street SW, Rochester, MN 55905, USA; 3 Department of Biochemistry and Molecular Biology, Mayo Clinic, 200 First Street SW, Rochester, MN 55905, USA; 4 Division of Medical Oncology, Department of Oncology, Mayo Clinic, 200 First Street SW, Rochester, MN 55905, USA; 5 Division of Biomedical Statistics and Informatics, Department of Health Sciences Research, Mayo Clinic, 200 First Street SW, Rochester, MN 55905, USA; 6 RIKEN Center for Genomic Medicine, 1-7-22 Suehiro-cho, Tsurumi-ku, Yokohama City, Kanagawa 230-0045, Japan; 7 Massachusetts General Hospital Cancer Center, Harvard University, 55 Fruit Street, Boston, MA 02114, USA

## Abstract

**Introduction:**

Arthralgias and myalgias are major side effects associated with aromatase inhibitor (AI) therapy of breast cancer. In a recent genome-wide association study, we identified SNPs - including one that created an estrogen response element near the 3' end of the T-cell leukemia 1A (*TCL1A*) gene - that were associated with musculoskeletal pain in women on adjuvant AI therapy for breast cancer. We also showed estrogen-dependent, SNP-modulated variation in *TCL1A *expression and, in preliminary experiments, showed that TCL1A could induce IL-17RA expression. In the present study, we set out to determine whether these SNPs might influence cytokine expression and effect more widely, and, if so, to explore the mechanism of TCL1A-related AI-induced side effects.

**Methods:**

The functional genomic experiments performed included determinations of TCL1A, cytokine and cytokine receptor expression in response to estrogen treatment of U2OS cells and lymphoblastoid cell lines that had been stably transfected with estrogen receptor alpha. Changes in mRNA and protein expression after gene knockdown and overexpression were also determined, as was NF-κB transcriptional activity.

**Results:**

Estradiol (E2) increased TCL1A expression and, in a *TCL1A *SNP-dependent fashion, also altered the expression of IL-17, IL-17RA, IL-12, IL-12RB2 and IL-1R2. TCL1A expression was higher in E2-treated lymphoblastoid cell lines with variant SNP genotypes, and induction of the expression of cytokine and cytokine receptor genes was mediated by TCL1A. Finally, estrogen receptor alpha blockade with ICI-182,780 in the presence of E2 resulted in greatly increased NF-κB transcriptional activity, but only in cells that carried variant SNP genotypes. These results linked variant *TCL1A *SNP sequences that are associated with AI-dependent musculoskeletal pain with increased E2-dependent TCL1A expression and with downstream alterations in cytokine and cytokine receptor expression as well as NF-κB transcriptional activity.

**Conclusions:**

SNPs near the 3' terminus of *TCL1A *were associated with AI-dependent musculoskeletal pain. E2 induced SNP-dependent TCL1A expression, which in turn altered IL-17, IL-17RA, IL-12, IL-12RB2, and IL-1R2 expression as well as NF-κB transcriptional activity. These results provide a pharmacogenomic explanation for a clinically important adverse drug reaction as well as insights into a novel estrogen-dependent mechanism for the modulation of cytokine and cytokine receptor expression.

## Introduction

The introduction of aromatase inhibitors (AIs), drugs that block the enzyme that synthesizes estrogens, to treat women with estrogen receptor (ER)-positive breast cancer marked a significant advance in the treatment of this disease, with a reduction in recurrence of approximately 50% [1]. However, AI therapy can also result in drug-induced musculoskeletal pain as a major side effect that can result in the termination of AI therapy [2]. For example, in the Arimidex, Tamoxifen, Alone or in Combination breast cancer clinical trial, up to 28% of women treated with AIs developed musculoskeletal pain, and approximately 10% discontinued therapy because of this adverse drug reaction [3]. Changes in circulating estrogen levels in women have long been associated with musculoskeletal symptoms. Arthritis of the menopause was described by Cecil and Archer over 85 years ago [4], and joint pain was a major complaint among participants in the Women's Health Initiative study after the withdrawal of estrogen therapy [5].

We recently performed a case-control genome-wide association study (GWAS) of participants in the NCIC-CTG MA.27 clinical trial of AI adjuvant therapy in postmenopausal women with ER-positive breast cancer in an attempt to identify biomarkers and define mechanisms responsible for musculoskeletal pain associated with pharmacologic blockade of estrogen synthesis. That GWAS identified a SNP signal on chromosome 14 that mapped near the 3' end of the T-cell leukemia 1A (*TCL1A*) gene [6], and the SNP with the lowest *P *value (rs11849538, *P *= 6.67 × 10^-7^) created a functional estrogen response element (ERE). We also observed that TCL1A expression was induced by estrogen exposure, and that it was significantly elevated in lymphoblastoid cell lines (LCLs) that carried variant sequences for the chromosome-14 SNPs; that is, in cell lines with DNA encoding the SNP-related ERE. The present study was performed to pursue possible mechanisms by which these SNPs might be associated with musculoskeletal pain in response to reduced estrogen levels during AI therapy, mechanisms that might also have broader implications for the role of estrogens in musculoskeletal pain [6].

TCL1A is a member of a TCL1 family of proteins that includes TCL1A, TCL1B and TCL6 [7]. This protein is expressed in activated T lymphocytes and B lymphocytes as well as thymocytes, can interact with Akt and can enhance Akt kinase activity [8-11], but little is otherwise known about TCL1A function. In follow-up of our original GWAS, we reported that TCL1A expression was estrogen dependent and was correlated with expression of the cytokine receptor IL-17RA [6]. In the present study, we set out to determine whether TCL1A expression - expression that is estrogen dependent but is altered by the SNPs that were associated with AI-induced musculoskeletal pain - might also be associated with variation in the expression of other cytokines and/or cytokine receptors. Many of the experiments described subsequently were performed with U2OS cells because those cells express TCL1A and have been stably transfected with ERα, and with a powerful genomic data-rich LCL model system that includes cell lines with known *TCL1A *SNP genotypes. The availability of these LCLs, also stably transfected with ERα, made it possible for us to link the SNPs that we observed during the clinical GWAS for AI-induced musculoskeletal pain with variation in the expression of a series of cytokine and cytokine receptor genes that have been implicated in arthritis. Specifically, we observed, as described in detail subsequently, that estrogen-dependent, SNP-modulated expression of TCL1A is not only associated with variation in IL-17RA expression but also with the expression of IL-17, IL-1R2, IL-12 and IL-12RB2 as well as variation in NF-κB transcriptional activity.

## Materials and methods

### Human Variation Panel lymphoblastoid cell lines

The Human Variation Panel of LCLs from 100 healthy European-American, 100 African-American and 100 Han Chinese-American subjects was obtained from the Coriell Institute (Camden, NJ, USA). These cell lines were generated from blood samples obtained by the National Institute of General Medical Sciences. We genotyped DNA from these cell lines for genome-wide SNPs using the Illumina 550K and 510S SNP BeadChip (Illumina, San Diego, CA, USA). The Coriell Institute also genotyped DNA from the same cell lines using the Affymetrix SNP Array 6.0 (Affymetrix, Santa Clara, CA, USA) for a total of ~1.3 million unique SNPs per cell line [12]. We also generated basal Affymetrix U133 2.0 Plus GeneChip expression array data for all of the cell lines. This LCL genomic model system has been described in detail elsewhere [12]. The microarray data and SNP data for these LCLs have been deposited in the NCBI Gene Expression Omnibus [13] under SuerSeries [GEO:GSE24277].

### Lymphoblastoid cell line transfection and culture

Three of the European-American Human Variation Panel LCLs with variant genotypes for the chromosome-14 SNPs rs7158782, rs7159713, rs2369049 and rs11849538, and three with wild type (WT) sequences were stably transfected with a pcDNA4.1-ERα construct provided by Dr Thomas Spelsberg (Mayo Clinic, Rochester, MN, USA). Genotypes for the rs11849538 SNP in the cell lines were confirmed by performing the PCR with genomic DNA as the template using the following primers: forward, 5'-GTGACAAGAAAGCTGTGGACTAGAGACACA-3'; and reverse, 5'-TTGGAGGCATACGTTGAGAACCATTGGAGTAA-3'. Genotypes for the other three chromosome-14 SNPs in these cells had been determined during GWAS genotyping. These six stably transfected LCLs were cultured with or without estradiol (E2), as described previously [6]. In some experiments, the ERα inhibitor ICI-182,780 (Tocris, Baldwin, MO, USA) was added to culture medium containing 0.01 nM E2 for an additional 24 hours after the initial 24 hours at final E2 concentrations of 10^-10^, 10^-9^, 10^-8 ^and 10^-7 ^μM, and total RNA was isolated from the cells with the RNeasy mini kit (Qiagen, Valencia, CA, USA). Two hundred nanograms of this RNA was then used to perform quantitative RT-PCR with appropriate primers. Expression levels were normalized on the basis of ERα expression in each cell line.

### NF-κB transcriptional activity

U2OS-ERα cells [14] were seeded in triplicate in 12-well cell culture plates, with 10^5 ^cells/well. After 24 hours, the cells were transfected using Lipofectamine 2000 (Invitrogen, Grand Island, NY, USA) with two plasmids; one encoding an NF-κB promoter-luciferase construct (SABioscience, Foster City, CA, USA), and the other encoding a Renilla luciferase vector (Promega, Madison, WI, USA). Co-transfection with the Renilla construct made it possible to correct for possible variation in transfection efficiency. Transfected cells were incubated overnight and were then grown for 24 hours in DMEM with 5% charcoal-stripped FBS, followed by incubation in DMEM without FBS for 24 hours, either with ethanol or with 0.1 nM E2 dissolved in ethanol. The cells were then harvested and analyzed for luciferase activity (Promega).

The six LCLs with differing genotypes for the four chromosome-14 SNPs that had been stably transfected with ERα were also transiently transfected with 2 μg NF-κB reporter plasmid and 500 ng Renilla luciferase or 2 μg empty reporter plasmid plus 500 ng Renilla luciferase construct. Specifically, 2 × 10^6 ^cells were suspended in Cell Line Nucleofector Kit V solution (Lonza, Cologne, Germany) with 2 μg purified NF-κB reporter plasmid and 500 ng Renilla reporter plasmid, and were electroporated with the T-030 program using the Amaxa Nucleofector II (Amaxa Biosystems, Gaithersburg, MD, USA). Cells from six electroporation procedures per LCL were pooled to obtain 1.2 × 10^7 ^cells. Electroporated cells were then plated in RPMI 1640 medium supplemented with 15% FBS, were allowed to recover from electroporation for 24 hours, and were cultured for 24 hours in RPMI 1640 media containing 5% (vol/vol) charcoal-stripped FBS, followed by incubation for 24 hours in the same media containing increasing concentrations of E2. At that time, cells treated with 0.01 nM E2 were exposed to increasing concentrations of ICI-182,780 for a final 24 hours. Luciferase assays were performed, and those values were corrected for possible variation in transfection efficiency by use of the Renilla luciferase values.

### Western blot analysis

Proteins were isolated from U2OS-ERα cells after lysis with CelLytic M Cell Lysis buffer (Sigma-Aldrich, St. Louis, MO, USA) and were subjected to electrophoresis on 15% SDS-PAGE gels, followed by transfer to polyvinylidene fluoride membranes. The polyvinylidene fluoride membranes were probed with appropriate antibodies, and protein bands were visualized using enhanced chemiluminescence (Thermo Scientific, Rockford, IL, USA).

## Results

### NCIC-CTG MA.27 genome-wide association study

A brief description of the MA.27 breast cancer clinical trial GWAS is necessary prior to describing the functional implications of the chromosome-14 SNPs that were associated with musculoskeletal pain during that study. MA.27 is a large adjuvant AI clinical trial of patients with postmenopausal ER-positive breast cancer who were treated with one of two AIs, anastrozole or exemestane. There was no difference between the two drugs in breast cancer recurrence [15,16]. For both drugs, however, the primary reason for discontinuing drug therapy was the occurrence of musculoskeletal pain. We genotyped DNA samples from women enrolled in the MA.27 trial in an attempt to identify SNPs associated with musculoskeletal pain after AI therapy [17].

The three GWAS SNPs with the lowest *P *values (rs7158782, rs7159713 and rs2369049) mapped to chromosome 14, and imputation followed by genotyping showed that one of the imputed SNPs (rs11849538) had an even lower *P *value (*P *= 6.67 × 10^-7^; odds ratio = 2.21) than the SNPs genotyped using the GWAS platform. All four of the SNPs with the lowest *P *values were in linkage disequilibrium, and all four mapped close to *TCL1A*, with rs11849538 (the imputed SNP with the lowest *P *value) located only 926 base pairs 3' to the *TCL1A *gene [6]. That SNP created a functional ERE. We also reported that estrogens induced TCL1A expression; that estrogen induction of TCL1A expression was significantly greater in LCLs that carried variant rather than WT SNP genotypes; and, finally, that TCL1A appeared to upregulate expression of the cytokine receptor gene *IL-17RA*. The present study was designed to move beyond these initial, preliminary functional observations. The results described subsequently link variation in estrogen-dependent TCL1A expression to variation in the expression of a series of genes encoding cytokines and cytokine receptors that are known to play a role in musculoskeletal pathophysiology, suggesting the existence of a novel estrogen-dependent, TCLIA-mediated mechanism for the regulation of cytokine expression that was uncovered by exposure to AI therapy.

### SNP-dependent regulation of TCL1A, IL-17RA, IL-17, IL-1R2, IL-12 and IL-12RB2 expression

As a first step in studying the possible relationship of the SNPs near *TCL1A *with the expression of cytokines and cytokine receptors, we took advantage of the Human Variation Panel LCL model system to ask whether variation in TCL1A mRNA expression might be associated with variation in cytokine or cytokine receptor expression in these cells. It was important that we use this cell system because genome-wide genotypes were known for all of these LCLs, so they could be used to evaluate the effect of the SNPs discovered during the clinical GWAS. There was a significant correlation between TCL1A expression and those of a series of cytokine receptor genes in the 300 cell lines included in this model system. All of the correlations of cytokine receptor expression with expression of TCL1A that had *P *≤ 10^-9 ^in the Human Variation Panel are listed in Table 1.

**Table 1:** Correlation of T-cell leukemia 1A expression with cytokine receptor expression in lymphoblastoid cell lines

Gene	Gene name	*P *value	Spearman ρ
*IL-13RA1*	IL-13 receptor, alpha 1	3.16 × 10^-14^	-0.428
*IL-18R1*	IL-18 receptor 1	2.77 × 10^-13^	-0.409
*IL-1R2*	IL-1 receptor, type 2	1.73 × 10^-11^	-0.384
*IL-17RA*	IL-17 receptor A	1.92 × 10^-10^	0.365
*IL-12RB2*	IL-12 receptor, beta 2	4.84 × 10^--9^	-0.337

Correlation of T-cell leukemia 1A expression with cytokine receptor expression in 300 lymphoblastoid cell lines from subjects of three different ethnicities.

**Figure 1:**
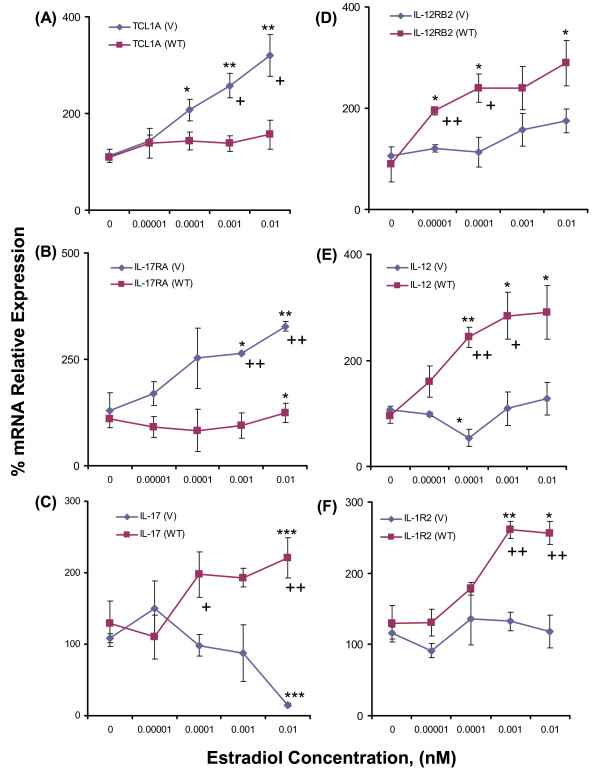
**Effect of estradiol concentration on mRNA expression for cytokine receptors in lymphoblastoid cell line SNPs**. Effect of estradiol (E2) concentration on mRNA expression for **(A) **T-cell leukemia 1A **(**TCL1A), **(B) **IL-17RA, **(C) **IL-17, **(D) **IL-12RB2, **(E) **IL-12 and **(F) **IL-1R2 in Human Variation Panel lymphoblastoid cell lines with wild type (WT) (*n *= 3) or variant (V) (*n *= 3) SNP sequences. *P < 0.05 and **P < 0.001 compared with zero E2, ^+^*P *< 0.05 and ^++^*P *< 0.01 for differences between WT and V SNP genotypes at the same E2 concentrations.

We next determined the effect of estrogen-dependent TCL1A expression in LCLs with variant or WT chromosome-14 SNPs on the expression of these cytokine receptors and their ligands. Specifically, we tested the possible relationship of change in TCL1A expression in response to increasing concentrations of E2 with those for all of the cytokine receptors listed in Table 1 as well as their ligands. We found that the expression of TCL1A, IL-17RA, IL-17, IL-1R2, IL-12RB2 and IL-12 were all significantly altered in a SNP-dependent fashion by exposure of LCLs with known genotypes for the chromosome-14 SNPs to increasing concentrations of E2 (Figure 1). Specifically, there was increased expression of both TCL1A and IL-17RA in cells with variant SNP genotypes. In contrast, there was greater expression in cells with WT than in those with variant SNP alleles for IL-17, IL-12, IL-1R2 and IL-12RB2. These differences were confirmed and explored in the course of subsequent experiments. Furthermore, as described subsequently, knockdown or overexpression of TCL1A also demonstrated that TCL1A induction was upstream of changes in cytokine receptor or cytokine gene expression.

### TCL1A knockdown and overexpression

**Figure 2:**
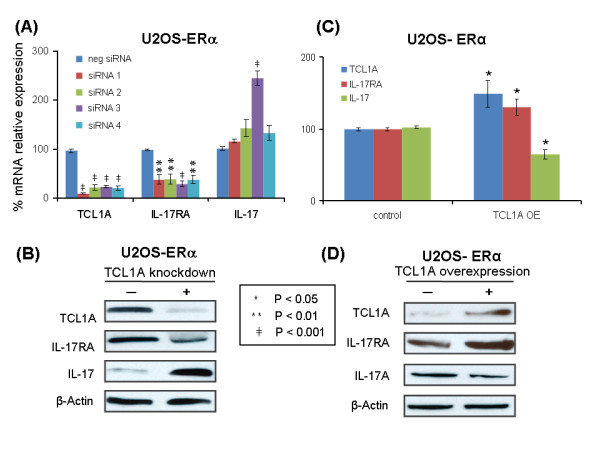
**T-cell leukemia 1A knockdown and overexpression and IL-17RA and IL-17 expression**. **(A) **Relative T-cell leukemia 1A (TCL1A), IL-17RA and IL-17 mRNA expression levels in U2OS-estrogen receptor alpha (ERα) cells after TCL1A was knocked down using four different specific siRNAs. **(B) **Western blots of TCL1A, IL-17RA, IL-17 and β-actin, with or without TCL1A knockdown in U2OS-ERα cells. **(C) **Relative TCL1A, IL-17RA and IL-17 mRNA expression levels after TCL1A overexpression (OE) in U2OS-ERα cells. **(D) **Western blots of TCL1A, IL-17RA, IL-17 and β-actin, with or without TCL1A overexpression in U2OS-ERα cells. All data are the mean ± standard error of the mean of triplicate determinations.

To determine whether correlations between the expression of TCL1A and those of cytokines and cytokine receptors shown in Figure 1 might represent a causal relationship, we next knocked down and overexpressed TCL1A and tested the effect on the expression of IL-17RA, IL-17, IL-1R2, IL-12 and IL-12RB2. Initially, we tested IL-17RA and IL-17 by performing quantitative RT-PCR and western blot analyses after TCL1A knockdown or overexpression. Those experiments were performed using U2OS-ERα cell lines because these cells express TCL1A - making it possible to determine the effect of knockdown - and also because they are stably transfected with ERα, making it possible to test the effect of estrogen exposure. Knockdown was performed with four different TCL1A siRNAs (Qiagen, Valencia, CA, USA), resulting in significantly decreased IL-17RA mRNA expression for all four siRNAs tested, with either no change or a significant increase in IL-17 expression (Figure 2A). Overexpression of TCL1A resulted in increased IL-17RA mRNA expression and significantly decreased expression of IL-17 (Figure 2C). Results of western blot analysis paralleled the mRNA changes (Figure 2B, D).

**Figure 3:**
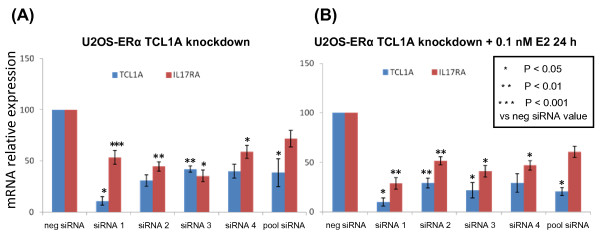
**Estradiol upregulates T-cell leukemia 1A and IL-17RA in series**. **(A) **T-cell leukemia 1A (TCL1A) and IL-17RA expression after TCL1A was knocked down in U2OS-estrogen receptor alpha (ERα) cells by four different TCL1A-specific siRNAs and by a pool of the four siRNAs. **(B) **TCL1A and IL-17RA expression after TCL1A was knocked down by four different TCL1A-specific siRNAs and a pool of the four siRNAs with an additional 24 hours exposure to 0.1 nM estradiol (E2). All values represent the mean ± standard error of the mean of triplicate determinations.

Having observed that TCL1A and IL-17RA were upregulated by E2 (Figure 1), we wanted to determine whether E2 upregulated TCL1A and IL-17RA in parallel or in series; that is, whether TCL1A was upstream of IL-17RA - as suggested by the effect of the SNPs on estrogen-dependent cytokine expression (Figure 1). To test the hypothesis that TCL1A might be upstream of cytokine expression, we knocked down TCL1A using the same four siRNAs used to perform the experiments shown in Figure 2A, with or without the addition of 0.1 nM E2, and then assayed both TCL1A and IL-17RA mRNA expression by quantitative RT-PCR. When TCL1A was knocked down, IL-17RA expression also decreased (shown in Figure 3A). Furthermore, when 0.1 nM E2 was added after TCL1A knockdown, IL-17RA mRNA expression was no different from that observed in the absence of estrogen (Figure 3B) - indicating that E2 did not directly increase IL-17RA expression, but rather that TCL1A was upstream of IL-17RA.

**Figure 4:**
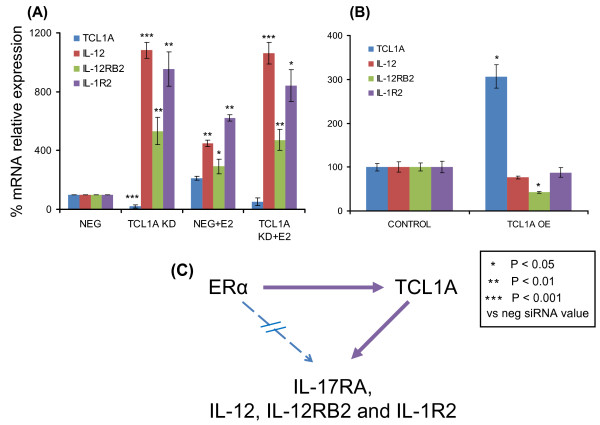
**Estradiol upregulates T-cell leukemia 1A upstream of IL-12, IL-12RB2 and IL-1R2**. **(A) **T-cell leukemia 1A (TCL1A), IL-12, IL-12RB2 and IL-1R2 expression in U2OS-estrogen receptor alpha (ERα) cells after TCL1A was knocked down (KD) by an siRNA pool, with or without subsequent exposure to 0.1 nM estradiol (E2) for 24 hours. **(B) **TCL1A, IL-12, IL-12RB2 and IL-1R2 expression in U2OS-ERα cells after TCL1A overexpression (OE). All values are the mean ± standard error of the mean of triplicate determinations. **(C) **Diagrammatic representation of the sequential E2 upregulation of TCL1A followed by altered cytokine or cytokine receptor expression.

To determine whether the other cytokines and cytokine receptors shown in Figure 1 behaved in a similar fashion, we knocked down and overexpressed TCL1A and performed quantitative RT-PCR, as we had for IL-17RA. When we knocked down TCL1A using an siRNA pool (Dharmacon, Lafayette, CA, USA), either with or without 24 hours of exposure to 0.1 nM E2, TCL1A knockdown resulted in significant increases in IL-12, IL-12RB2 and IL-1R2 mRNA expression (Figure 4A). Furthermore, after TCL1A knockdown, addition of 0.1 nM E2 did not change the expression of these cytokines and cytokine receptors beyond that seen with TCL1A knockdown alone (Figure 4A), exactly as we had observed for IL-17RA. Conversely, overexpression of TCL1A resulted in a 24% decrease in IL-12, a 58% decrease in IL-12RB2 and a 13% decrease in IL-1R2 mRNA expression in the same cells (Figure 4B). These results were compatible with the conclusion that E2 upregulates TCL1A expression and that TCL1A upregulation then increases IL-17RA expression, but results in decreased IL-12, IL-12RB2 and IL-1R2 expression in this cell line (depicted schematically in Figure 4C). However, even though this series of experiments indicated that TCL1A, in a SNP-dependent fashion, could mediate E2-dependent regulation of the expression of selected cytokine receptors and cytokines, they did not clearly explain how reduced estrogen concentrations might result in inflammation or musculoskeletal pain. We therefore also determined whether TCL1A might influence the transcriptional activity of NF-κB, a known mediator of joint inflammation [18].

### TCL1A downregulation of NF-κB transcriptional activity

NF-κB is activated in the joints of patients with many rheumatologic diseases [19], and IL-17 is known to influence NF-κB transcriptional activity [20]. TCL1A is an Akt co-activator [21], and activation of the phosphoinositide 3-kinase/Akt pathway negatively regulates NF-κB [22]. An effect of TCL1A on NF-κB could therefore potentially contribute to the relationship of TCL1A to musculoskeletal pain in patients treated with AIs, so we attempted to determine whether TCL1A might alter NF-κB transcriptional activity. NF-κB proteins are sequestered in the cell by binding to a family of inhibitory IκB proteins [23]. A variety of stimuli, including many cytokines, can lead to IκB phosphorylation catalyzed by IKK [24].

**Figure 5:**
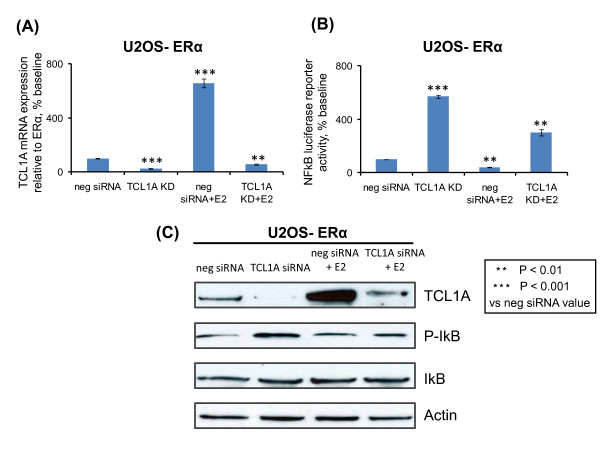
**T-cell leukemia 1A knockdown and NF-κB transcriptional activity**. **(A) **T-cell leukemia 1A (TCL1A) expression in U2OS-estrogen receptor alpha (ERα) cells before and after siRNA knockdown (KD), with or without the addition of 0.1 nM estradiol (E2) for an additional 24 hours. **(B) **NF-κB transcriptional activity after co-transfection with an NF-κB reporter construct together with TCL1A-negative siRNA or TCL1A-specific siRNA. Forty-eight hours after transfection, the cells were exposed to 0.1 nM E2 for an additional 24 hours. All values are the mean ± standard error of the mean of triplicate determinations. **(C) **Western blots for TCL1A, phosphorylated IκB, IκB and actin after TCL1A knockdown in U2OS-ERα cells, with or without the addition of 0.1 nM E2 for an additional 24 hours.

To determine whether TCL1A might influence NF-κB transcriptional activity, we transfected U2OS-ERα cells with an NF-κB reporter construct. TCL1A knockdown in those cells to 22% of baseline (Figure 5A) increased NF-κB transcriptional activity by 5.7-fold (Figure 5B). When the cells were incubated for 24 hours with 0.1 nM E2, however, TCL1A mRNA increased 6.9-fold and NF-κB activity decreased to 38% of the value after exposure to negative siRNA. Incubation with 0.1 nM E2 after knockdown of TCL1A resulted in TCL1A mRNA levels that were 56% of those after negative siRNA treatment (Figure 5A), while NF-κB activity in the same cells increased threefold (Figure 5B). Western blot analysis confirmed these results as well as the association of these alterations in TCL1A expression with those of phosphorylated IκB in the same cells (Figure 5C). These results suggested that a decrease in TCL1A expression after estrogen withdrawal (for example, after treatment with ER antagonists or AIs) might increase NF-κB transcriptional activity. The next experiment was performed in an effect to determine the effect of increasing concentrations of E2 or of E2 withdrawal on NF-κB transcriptional activity, as well as the effect of the chromosome-14 SNPs near *TCL1A *on the response to those changes in estrogen effect.

### *TCL1A *SNPs, estrogen-dependent TCL1A expression and NF-κB activity

To determine the effect of the SNPs near *TCL1A *on estrogen withdrawal and on NF-κB transcriptional activity, we treated Human Variation Panel LCLs - three with WT SNP genotypes and three with variant SNP genotypes, all stably transfected with ERα - with low concentrations of estrogen, followed by ER blockade to replicate the estrogen withdrawal that occurs during AI therapy. Specifically, these six LCLs were treated with increasing concentrations of E2, followed by blockade of the ER with ICI-182,780 [14], still in the presence of 0.01 nM E2. The quantitative RT-PCR and NF-κB reporter assays were then performed to determine whether the SNPs might influence estrogen-dependent TCL1A expression and/or NF-κB transcriptional activity.

**Figure 6:**
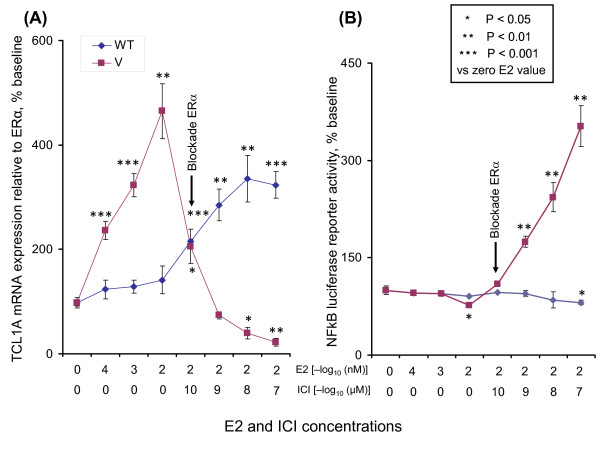
**SNP-related variation in T-cell leukemia 1A expression and NF-κB activity in lymphoblastoid cell lines**. SNP-related variation in T-cell leukemia 1A (TCL1A) expression and NF-κB transcriptional activity in three lymphoblastoid cell lines (LCLs) with variant (V) genotypes and three LCLs with wild type (WT) genotypes for the chromosome-14 SNPs after exposure to increasing concentrations of estradiol (E2), or E2 plus the estrogen receptor (ER) antagonist ICI-182,780. **(A) **Quantitative RT-PCR of TCL1A expression after exposure to increasing concentrations of E2 for 24 hours, followed by 0.01 nM E2 plus increasing concentrations of ICI-182,780 for an additional 24 hours. **(B) **The same six LCLs were transfected with an NF-κB reporter construct. After exposure to increasing concentrations of E2 for 24 hours, followed by 0.01 nM E2 plus increasing concentrations of ICI-182,780 for an additional 24 hours, luciferase activities were determined. All values represent the mean ± standard error of the mean of triplicate determinations.

When the LCLs were treated with increasing concentrations of E2 (0.00001 to 0.01 nM), concentrations similar to those found in the plasma of postmenopausal women [25], average *TCL1A *expression increased approximately five fold in LCLs with variant SNP genotypes (Figure 6A). In the three cell lines with the WT genotype, however, average TCL1A expression increased only approximately 40%, as expected on the basis of our previous results as shown in Figure 1[6]. To determine the effect of ER blockade in this setting, increasing concentrations (from 10^-10 ^to 10^-7 ^μM) of the ER antagonist, ICI-182,780, were then added to the cells in the presence of 0.01 nM E2. TCL1A expression in cells with the variant genotype decreased to levels well below baseline, while expression in cells with WT genotypes continued to increase to approximately 3.5-fold those of baseline (Figure 6A).

To determine whether the change in TCL1A expression that occurred in response to ER blockade might influence NF-κB transcriptional activity, NF-κB reporter gene assays were performed (Figure 6B) with the same samples shown in Figure 6A. NF-κB transcriptional activity did not differ greatly (from 70 to 100% of baseline) between cells with WT and variant genotypes as E2 concentrations increased from 0.00001 to 0.01 nM. In cells with variant SNP genotypes, however, the NF-κB activity rose over three fold when the ERα antagonist was added. This behavior contrasted with that of cell lines with the WT genotype, in which NF-κB activity decreased slightly (Figure 6B). The ICI-182,780 blockade of ERα therefore resulted in increased TCL1A-mediated stimulation of NF-κB transcriptional activity in cells with variant SNP genotypes, in contrast to the situation seen for cell lines with WT genotypes. This increase in NF-κB transcriptional activity after estrogen withdrawal may contribute to increased risk for the occurrence of musculoskeletal pain in subjects with variant SNP genotypes who have estrogen synthesis blocked with AIs.

## Discussion

The use of adjuvant AI therapy to treat ER-positive breast cancer patients represents a major advance in the treatment of that disease [26]. A recent report demonstrating that an AI (exemestane) was highly efficacious in preventing breast cancer highlights the importance of understanding mechanisms responsible for the musculoskeletal side effects of AI therapy [27]. These side effects limit patient adherence to therapy with this important class of drugs for either breast cancer treatment or prevention. Equally important, however, may be the fact that AI therapy represents a medically-indicated form of pharmacologic estrogen deprivation that might provide a window on mechanisms by which estrogen withdrawal can cause musculoskeletal symptoms. Our GWAS performed with DNA samples from patients enrolled in the MA.27 clinical trial identified four SNPs on chromosome 14 near the 3' end of *TCL1A *that were associated with increased risk for musculoskeletal adverse events in women receiving adjuvant AI therapy for the treatment of ER-positive breast cancer [6]. In the present study, we pursued those observations and linked E2-dependent induction of TCL1A to the expression of a series of cytokines and cytokine receptors, including IL-17RA, IL-17, IL-12RB2, IL-12 and IL-1R2, with SNP-dependent variation in this induction (Figure 1). Obviously, results obtained for other cell lines might identify additional or different cytokines/cytokine receptors, but the purpose of this study was to take the first step in the elucidation of a novel pathway for estrogen-dependent, *TCL1A *SNP-dependent regulation of cytokine and cytokine receptor expression. Our results also demonstrated a relationship between estrogen and SNP-dependent variation in TCL1A expression and NF-κB transcriptional activity (Figure 6).

TCL1A expression is associated with CD4^+ ^and CD8^+ ^T-cell activation through the phosphoinositide 3-kinase/Akt signaling pathway [28], and TCL1A enhances Akt kinase activity [29]. However, there have been no previous reports of the regulation of TCL1A by estrogens or of an association of TCL1A expression with cytokine or cytokine receptor expression. A recent study did report that another member of the TCL1 gene family, *TCL1B*, is E2-inducible because of an ERE located near the 3' end of that gene [30]. The SNP with the lowest *P *value in our GWAS, rs11849538, created an ERE near the 3' terminus of *TCL1A*, and cell lines that carried the variant SNP genotype displayed increased TCL1A expression after estrogen exposure (see Figures 1 and 6A).

In the present study, we found that increased expression of TCL1A upregulated IL-17RA expression and downregulated the expression of IL-17, IL-12, IL-12RB2 and IL-1R2 (Figure 1). IL-17 has been reported not only to drive the T-helper type 17 immune pathway, but also to regulate the T-helper type 1 pathway by decreasing IL-12 and IL-12RB2 subunit expression, especially in patients with rheumatoid arthritis [31]. The E2-dependent regulation of cytokine and cytokine receptor expression that is mediated by TCL1A might help explain the association of TCL1A with musculoskeletal symptoms in patients treated with AIs. TCL1A can also influence NF-κB transcriptional activity (Figure 6), suggesting that, after estrogen withdrawal, increased NF-κB activity might contribute to AI-induced musculoskeletal pain. We also showed that cell lines containing variant chromosome-14 SNP genotypes had significantly elevated TCL1A expression after exposure to increasing concentrations of estrogen (Figures 1 and 6A). After ER blockade with ICI-182,780, however, TCL1A expression dropped precipitously in LCLs with variant SNPs, while it was elevated in cells with the WT SNP genotypes (Figure 6A). Conversely, NF-κB transcriptional activity increased after ER blockade in cells carrying variant SNP genotypes (Figure 6B).

## Conclusions

In summary, this series of experiments - studies that began with a GWAS performed using DNA from women receiving AIs to treat breast cancer - may provide insight into mechanisms that relate AI-dependent estrogen withdrawal to musculoskeletal symptoms. AI therapy is designed to result in a striking decrease in estrogen synthesis and in decreased circulating estrogen levels [6]. As a result, treatment with AIs represents medically-indicated, pharmacologic estrogen deprivation - a therapeutic maneuver that might provide insight into the possible role of decreased circulating estrogens in musculoskeletal pathophysiology.

## Abbreviations

AI: aromatase inhibitor; DMEM: Dulbecco's modified Eagle's medium; E2: estradiol; ER: estrogen receptor; ERE: estrogen response element; FBS: fetal bovine serum; GWAS: genome-wide association study; IL: interleukin; IL-1R2: IL-1 receptor: type 2; IL-12RB2: IL-12 receptor: beta 2; IL-17RA: IL-17 receptor A; LCL: lymphoblastoid cell line; NF: nuclear factor; PCR: polymerase chain reaction; RT: reverse transcriptase; siRNA: small interfering RNA; SNP: single nucleotide polymorphism; TCL1A: T-cell leukemia 1A; WT: wild type.

## Competing interests

The authors declare that they have no competing interests.

## Authors' contributions

ML, LW, JNI, DJS, JRH, SNM, PEG, TM, MK, YN and RMW designed the research. ML, DJS, PEG, TM, MK, YN, NK and RMW performed the research. ML, LW, TB, DJS, SNM and RMW analyzed the data. ML, LW, TB, JNI, DJS, JRH, SNM, PEG and RMW wrote the paper. All authors read and approved the final manuscript.

## References

[B1] Del Mastro L Clavarezza M Venturini M Reducing the risk of distant metastases in breast cancer patients: role of aromatase inhibitors Cancer Treat Rev 2007 33 681 687 10.1016/j.ctrv.2007.07.014 17850976

[B2] Khan QJ O'Dea AP Sharma P Musculoskeletal adverse events associated with adjuvant aromatase inhibitors J Oncol 2010 in press pii: 654348 10.1155/2010/654348 PMC2943085 20871846

[B3] Burstein HJ Aromatase inhibitor-associated arthralgia syndrome Breast 2007 16 223 234 10.1016/j.breast.2007.01.011 17368903

[B4] Cecil RL Archer BH Arthritis and the menopause J Am Med Assoc 1925 84 75 79 10.1001/jama.1925.02660280001001

[B5] Brunner RL Aragaki A Barnabei V Cochrane BB Gass M Hendrix S Lane D Ockene J Woods NF Yasmeen S Stefanick M Menopausal symptom experience before and after stopping estrogen therapy in the Women's Health Initiative randomized, placebo-controlled trial Menopause 2010 17 946 954 10.1097/gme.0b013e3181d76953 20505547 PMC3770143

[B6] Ingle JN Schaid DJ Goss PE Liu M Mushiroda T Chapman JA Kubo M Jenkins GD Batzler A Shepherd L Pater J Wang L Ellis MJ Stearns V Rohrer DC Goetz MP Pritchard KI Flockhart DA Nakamura Y Weinshilboum RM Genome-wide associations and functional genomic studies of musculoskeletal adverse events in women receiving aromatase inhibitors J Clin Oncol 2010 28 4674 4682 10.1200/JCO.2010.28.5064 20876420 PMC3020700

[B7] Fu TB Virgilio L Narducci MG Facchiano A Russo G Croce CM Characterization and localization of the TCL-1 oncogene product Cancer Res 1994 54 6297 6301 7987816

[B8] Herling M Patel KA Weit N Lilienthal N Hallek M Keating MJ Jones D High TCL1 levels are a marker of B-cell receptor pathway responsiveness and adverse outcome in chronic lymphocytic leukemia Blood 2009 114 4675 4686 10.1182/blood-2009-03-208256 19770358 PMC2780304

[B9] Kunstle G Laine J Pierron G Kagami Si S Nakajima H Hoh F Roumestand C Stern MH Noguchi M Identification of Akt association and oligomerization domains of the Akt kinase coactivator TCL1 Mol Cell Biol 2002 22 1513 1525 10.1128/MCB.22.5.1513-1525.2002 11839817 PMC134690

[B10] Laine VJ Grass DS Nevalainen TJ Resistance of transgenic mice expressing human group II phospholipase A2 to *Escherichia coli *infection Infect Immun 2000 68 87 92 10.1128/IAI.68.1.87-92.2000 10603372 PMC97105

[B11] Pekarsky Y Hallas C Croce CM The role of TCL1 in human T-cell leukemia Oncogene 2001 20 5638 5643 10.1038/sj.onc.1204596 11607815

[B12] Niu N Qin Y Fridley BL Hou J Kalari KR Zhu M Wu TY Jenkins GD Batzler A Wang L Radiation pharmacogenomics: a genome-wide association approach to identify radiation response biomarkers using human lymphoblastoid cell lines Genome Res 2010 20 482 492 10.1101/gr.107672.110 PMC2963812 20923822

[B13] Gene Expression Omnibus http://www.ncbi.nih.gov/geo

[B14] Wu X Hawse JR Subramaniam M Goetz MP Ingle JN Spelsberg TC The tamoxifen metabolite, endoxifen, is a potent antiestrogen that targets estrogen receptor alpha for degradation in breast cancer cells Cancer Res 2009 69 1722 1727 10.1158/0008-5472.CAN-08-3933 19244106

[B15] Goss PE Ingle JN Chapman J-AW Ellis MJ Sledge GW Budd GT Rabaglio M Gelmon K Shepherd L Pritchard KI Final analysis of NCIC CTG MA.27: a randomized phase III trial of exemestane versus anastrozole in postmenopausal women with hormone receptor positive primary breast cancer Cancer Res 2010 70 24 Suppl 75s

[B16] Ryan PD Goss PE Adjuvant hormonal therapy in peri- and postmenopausal breast cancer Oncologist 2006 11 718 731 10.1634/theoncologist.11-7-718 16880231

[B17] Muslimani AA Spiro TP Chaudhry AA Taylor HC Jaiyesimi I Daw HA Aromatase inhibitor-related musculoskeletal symptoms: is preventing osteoporosis the key to eliminating these symptoms? Clin Breast Cancer 2009 9 34 38 10.3816/CBC.2009.n.006 19299238

[B18] Bokarewa M Nagaev I Dahlberg L Smith U Tarkowski A Resistin, an adipokine with potent proinflammatory properties J Immunol 2005 174 5789 5795 15843582 10.4049/jimmunol.174.9.5789

[B19] Okamoto T NF-κB and rheumatic diseases Endocr Metab Immune Disord Drug Targets 2006 6 359 372 17214582 10.2174/187153006779025685

[B20] Levin SD IL-17 receptor signaling: ubiquitin gets in on the act Sci Signal 2009 2 pe64 10.1126/scisignal.292pe64 19825826

[B21] Laine J Kunstle G Obata T Sha M Noguchi M The protooncogene TCL1 is an Akt kinase coactivator Mol Cell 2000 6 395 407 10.1016/S1097-2765(00)00039-3 10983986

[B22] Zhao L Lee JY Hwang DH The phosphatidylinositol 3-kinase/Akt pathway negatively regulates Nod2-mediated NF-κB pathway Biochem Pharmacol 2008 75 1515 1525 10.1016/j.bcp.2007.12.014 18243161

[B23] Kalaitzidis D Gilmore TD Transcription factor cross-talk: the estrogen receptor and NF-κB Trends Endocrinol Metab 2005 16 46 52 10.1016/j.tem.2005.01.004 15734144

[B24] Park KJ Krishnan V O'Malley BW Yamamoto Y Gaynor RB Formation of an IKKα-dependent transcription complex is required for estrogen receptor-mediated gene activation Mol Cell 2005 18 71 82 10.1016/j.molcel.2005.03.006 15808510

[B25] Ingle JN Buzdar AU Schaid DJ Goetz MP Batzler A Robson ME Northfelt DW Olson JE Perez EA Desta Z Weintraub RA Williard CV Flockhart DA Weinshilboum RM Variation in anastrozole metabolism and pharmacodynamics in women with early breast cancer Cancer Res 2010 70 3278 3286 10.1158/0008-5472.CAN-09-3024 20354183 PMC2855746

[B26] Ingle JN Overview of adjuvant trials of aromatase inhibitors in early breast cancer Steroids 2011 76 765 767 10.1016/j.steroids.2011.02.021 21382394

[B27] Richardson H Johnston D Pater J Goss P The National Cancer Institute of Canada Clinical Trials Group MAP.3 trial: an international breast cancer prevention trial Curr Oncol 2007 14 89 96 10.3747/co.2007.117 17593981 PMC1899358

[B28] Hoyer KK Herling M Bagrintseva K Dawson DW French SW Renard M Weinger JG Jones D Teitell MA T cell leukemia-1 modulates TCR signal strength and IFN-gamma levels through phosphatidylinositol 3-kinase and protein kinase C pathway activation J Immunol 2005 175 864 873 16002684 10.4049/jimmunol.175.2.864

[B29] Pekarsky Y Koval A Hallas C Bichi R Tresini M Malstrom S Russo G Tsichlis P Croce CM Tcl1 enhances Akt kinase activity and mediates its nuclear translocation Proc Natl Acad Sci USA 2000 97 3028 3033 10.1073/pnas.040557697 10716693 PMC16186

[B30] Badve S Collins NR Bhat-Nakshatri P Turbin D Leung S Thorat M Dunn SE Geistlinger TR Carroll JS Brown M Bose S Teitell MA Nakshatri H Subcellular localization of activated AKT in estrogen receptor- and progesterone receptor-expressing breast cancers: potential clinical implications Am J Pathol 2010 176 2139 2149 10.2353/ajpath.2010.090477 20228224 PMC2861080

[B31] Toh M-L Kawashima M Hot A Miossec P Miossec P Role of IL-17 in the Th1 systemic defects in rheumatoid arthritis through selective IL-12Rβ2 inhibition Ann Rheum Dis 2010 69 1562 1567 10.1136/ard.2009.111757 20542964

